# A rare presentation of solitary orbital cysticercosis

**DOI:** 10.1186/s12886-026-04768-y

**Published:** 2026-03-27

**Authors:** Ji-Hong Luo, Ming-Juan Li, Shu-Qiong Hu, Jun Fan, Jin Zhou, Chang-Mei Dong, Hui-Yu Jin

**Affiliations:** 1 Department of Ophthalmology, Hubei Provincial Hospital of Traditional Chinese Medicine, Affiliated Hospital of Hubei University of Chinese Medicine, Hubei Province Academy of Traditional Chinese Medicine, Wuhan, Hubei Province China; 2 Department of Ophthalmology, Huaihai Road Community Health Service Center, Qinhuai District, Nanjing, Jiangsu Province China; 3 Department of Ophthalmology, Jingzhou Hospital of Traditional Chinese Medicine, Jingzhou, Hubei Province China; 4 Department of Ophthalmology, Liyuan Hospital, Tongji Medical College, Huazhong University of Science and Technology, Wuhan, Hubei Province China

**Keywords:** Orbit, Cysticercosis, Orbital cysticercosis, Ocular cysticercosis, Myocysticercosis

## Abstract

A 68-year-old male presented with a three-month history of a painless, non-resolving mass in the left upper eyelid, unresponsive to a prior course of anti-inflammatory treatment. Orbital computed tomography (CT) revealed a well-defined, nodular soft-tissue density lesion in the lateral aspect of the orbit, adjacent to the orbital lobe of the lacrimal gland. The mass was successfully excised under local nerve block anesthesia. Histopathological examination confirmed the diagnosis of cysticercosis. Postoperative recovery was uneventful, with complete resolution of the eyelid deformity observed at the one-month follow-up.

**Clinical trial number** Not applicable.

## Introduction

Cysticercosis, caused by the larval stage of Taenia solium, represents a significant global parasitic burden, particularly in developing regions such as Mexico, Central and South America, Africa, Asia, and India [1]. Human infection typically occurs via the ingestion of tapeworm eggs from fecally contaminated food or water. Following eggs hatching in the intestine, the liberated larvae migrate via the bloodstream and lymphatic system, eventually encysting in various tissues to form cysticerci. Common sites of involvement include subcutaneous tissue, brain, and the eye [2]. Ocular cysticercosis most frequently affects the posterior segment (vitreous and subretinal space) and conjunctiva [3]. Orbital involvement, particularly isolated to the periocular tissues without intraocular extension, is a relatively unfrequent presentation. This report details a rare case of solitary orbital cysticercosis localized to the eyelid, diagnosed and managed in our institution in September 2024.

## Case report

A 68-year-old male farmer was referred to our hospital for evaluation of a painless mass in his left upper eyelid, which had been present for three months.

### History of present illness

The mass was not associated with pain, ptosis, redness, visual disturbance, or restriction of ocular motility. Prior to referral, a one-week course of oral anti-inflammatory medication administered at a local clinic failed to reduce the size of the mass. Following the outpatient diagnosis of a “left orbital mass,” the patient was admitted to our hospital for definitive diagnosis and surgical intervention.

The patient’s past medical, surgical, family, and social histories were unremarkable. However, it was worth mentioned that the patient recalled, after the surgery, a possible consumption of “rice pork”, a type of pork known to be a potential source of parasitic infection. Systemic review revealed no constitutional symptoms such as fever or weight loss. Upon admission, his general physical and vital signs were within normal limits.

### Ocular examination

Visual Acuity: 0.6 (Snellen) in both eyes (OD & OS).

Intraocular Pressure (IOP): 15mmHg OD and 17mmHg OS.

External & palpation examination: A well-defined, painless, non-tender, mobile mass (approximately 2*4 cm) was palpated in the left upper eyelid, near the supraorbital rim. Slit-lamp biomicroscopy of left eye showed conjunctiva and cornea clear, the anterior chamber with normal depth and quiet, the pupil round, approximately 3 mm in diameter, and reactive to light, and the lens opaque. Fundus examination revealed a clear optic disc and flat retina, though the macular reflex was dull.

Unremarkable anterior and posterior segment findings were spotted for right eye.

### Imaging findings

**Fig. 1:**
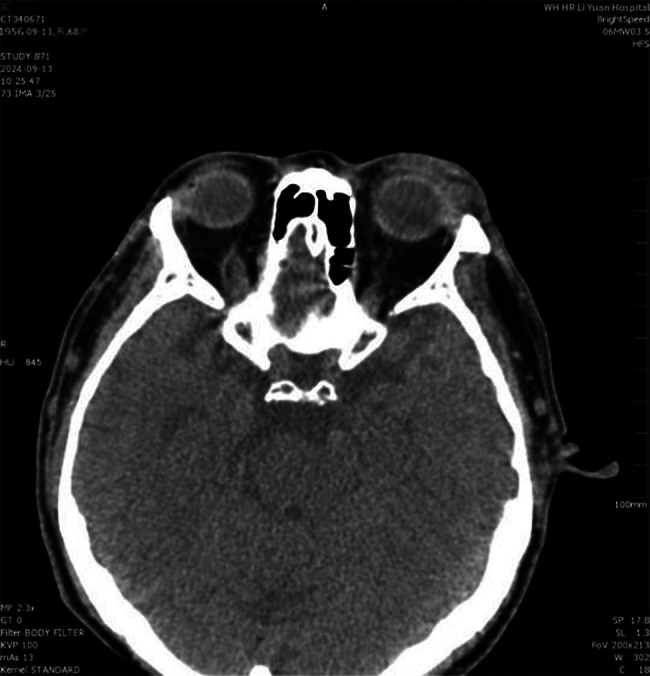
Axial CT scan revealed a well-circumscribed, nodular soft-tissue density lesion in the lateral aspect of the orbit, adjacent to the orbital lobe of the lacrimal gland

An orbital CT scan revealed a well-circumscribed, nodular soft-tissue density lesion in the lateral aspect of the orbit, adjacent to the orbital lobe of the lacrimal gland (Fig. 1).

#### Surgical procedure

**Fig. 2:**
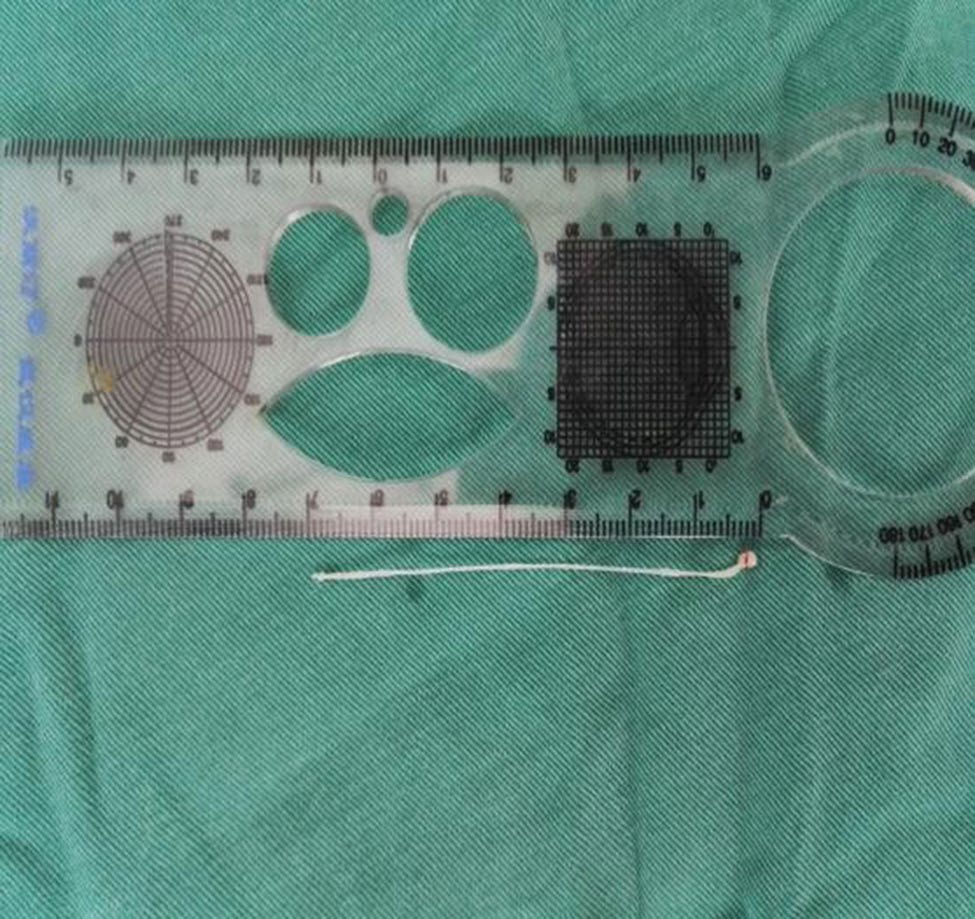
A viable white cysticercus larva was successfully removed from the orbit under local nerve block anesthesia

The patient underwent surgical excision of the left orbital mass under local nerve block anesthesia. A horizontal skin incision was made along the edge of the palpable lesion. The subcutaneous tissue, superior preseptal orbicularis muscle, and the septum were incised sequentially, revealing a well-defined cystic mass. The cyst wall was carefully incised, revealing a viable, white, cysticercus larva within, which was completely removed in toto (Fig. 2). The surrounding necrotic tissue was debrided. Hemostasis was achieved, and the wound was closed in layers. A small drain was placed subcutaneously, and antibiotic ointment was applied. The operative site was dressed with a sterile gauze pad.

### Postoperative course and outcome

**Fig. 3:**
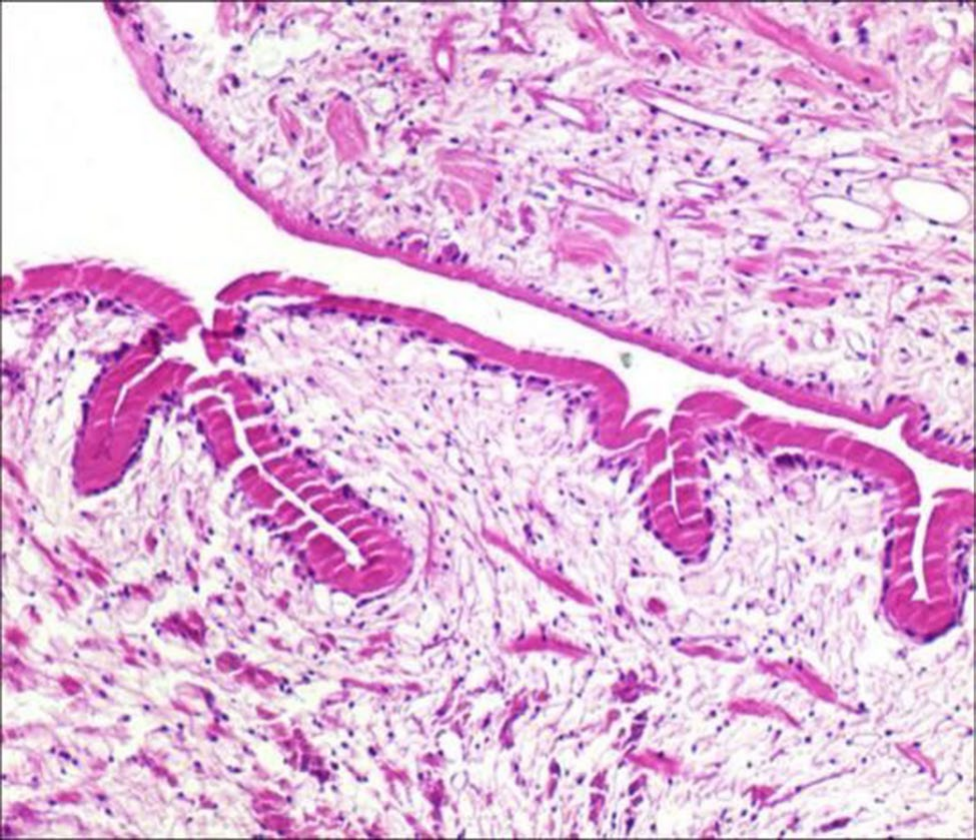
The findings included a cysticercus larva encapsulated by fibrous tissue, surrounded by a chronic inflammatory infiltrate composed of lymphocytes, plasma cells, and eosinophils

Pathological Diagnosis: Histopathological examination of the excised specimen confirmed the diagnosis of cysticercosis. The findings included a cysticercus larva within a fibrous capsule, surrounded by a chronic inflammatory infiltrate composed of lymphocytes, plasma cells, and eosinophils (Fig. 3).

Postoperative Medical Management: The patient received a 3-day course of intravenous antibiotics and corticosteroids. Empiric antibiotics were administered to prevent secondary bacterial infection, while corticosteroids were given to control postoperative inflammation and the inflammatory response to potential antigen release from the cyst. Antiparasitic therapy was not initiated due to the absence of clinical evidence of systemic parasitic involvement.

Clinical Follow-up: At the one-month postoperative visit, the mass had resolved completely. Visual acuity in the left eye improved to 0.8. Ophthalmic examination revealed no abnormalities.

Imaging Follow-up: A follow-up orbital CT scan demonstrated postoperative changes, including soft tissue thickening in the left upper eyelid, the globe contour was preserved, and no intraocular abnormalities or signs of residual disease were observed.

## Discussion

Ocular cysticercosis can affect any ocular or periocular tissue. The most common sites of involvement are the vitreous and subretinal spaces, while isolated orbital or adnexal involvement is relatively rare. Regarding orbital cysticercosis, a retrospective case series conducted between 2008 and 2018 reported that 90.16% (55/61) of patients had myocysticercosis, with the inferior rectus being the most commonly involved muscle, while the remaining 9.83% (6/61) had orbital cysticercosis without extraocular muscle involvement [6]. The clinical manifestations and severity of disease are primarily determined by the location, size, number of cysts, and their relationship to critical adjacent structures [4]. The pathogenesis of orbital cysticercosis involves a complex host immune response. The cysticerci release metabolic products and heterologous proteins, which diffuse into surrounding tissues and provoke a localized chronic granulomatous inflammation. Over time, this process leads to fibrous tissue proliferation, remodeling, and potential adhesion to vital structures such as extraocular muscles, the optic nerve, and periosteum, ultimately causing mechanical and inflammatory damage [5]. Clinical findings alone can be non-specific. Diagnosis relies on a combination of clinical suspicion, imaging, and serology; High-resolution ultrasonography (USG), computed tomography (CT), and magnetic resonance imaging (MRI) are pivotal for visualizing the characteristic cystic lesion, often revealing a scolex, which is pathognomonic [6].

The management of ocular cysticercosis is guided by the location of the parasite. For intraocular cysts, prompt surgical removal is the standard approach to eliminate the parasite, terminate the inflammatory stimulus, and prevent irreversible vision loss; In contrast, extraocular muscular or retro-orbital cysticercosis is often primarily managed with systemic antihelminthic therapy (e.g., albendazole), sometimes combined with corticosteroids to mitigate the inflammatory reaction [1, 7, 8, 10]. Both medical and surgical therapies can result in residual deficits, such as ocular movement restriction, diplopia, and strabismus. In orbital cysticercosis, surgical treatment can be considered as an alternative option. It has recently been advocated for subconjunctival and eyelid cysticercosis, as well as for specific cases requiring early intervention [6, 9, 10]. Careful dissection is paramount in all surgical procedures: while the parasite and necrotic debris should be completely excised, adherent fibrous scar tissue around functional structures should be preserved to avoid iatrogenic injury.

In our case, the surgical intervention achieved favorable outcomes, including successful removal of the cyst and a one-line improvement in visual acuity, a gain attributable to the resolution of the localized swelling induced by the lesion’s inflammatory response. Despite these positive results, however, we acknowledge several limitations in this case report. First, the absence of preoperative MRI is a limitation. Although an MRI was considered due to the atypical findings on CT, in retrospect, our initial low clinical suspicion for a complex orbital lesion led us to erroneously forgo it. We now recognize that MRI would have provided crucial additional information regarding the cyst’s character, which could have altered our preoperative planning. Second, a parasitic etiology was not sufficiently considered in the preoperative period, due to our narrow differential and limited awareness of its variable clinical and radiological presentations. Consequently, we did not pursue serologic testing or consult an infectious disease specialist for prophylactic antiparasitic therapy. This case is a powerful reminder that in the appropriate epidemiologic context, a parasitic lesion should remain on the differential for any cystic mass, even when classic imaging features are absent. Third, the intraoperative decision to incise the cyst, rather than perform a complete excision, represents another limitation. Given this diagnostic uncertainty, we incised the cyst wall to obtain tissue sample for histopathological confirmation in order to guide subsequent management. While the procedure was performed under protective measures, including betadine-soaked gauze packing and copious irrigation with povidone-iodine, this approach was nevertheless suboptimal, as it did not adequately address the potential risk of parasitic dissemination. This experience underscores that when a parasitic lesion cannot be confidently excluded preoperatively, a complete excision with spillage precautions should be the goal, rather than diagnostic incision.

## Conclusion

This case underscores that orbital cysticercosis should be considered in the differential diagnosis of solitary, treatment-resistant periocular masses, especially in endemic regions. Accurate diagnosis relies on a high index of clinical suspicion supported by characteristic imaging findings on ultrasound, CT, or MRI, with histopathological examination providing definitive confirmation. Treatment choice depends on the clinical scenario: while medical therapy is the mainstay, surgical excision remains an option for specific cases that require early intervention to remove the parasite and alleviate mass effect.

## Data Availability

Not applicable.
